# Impaired glucose tolerance and insulin resistance in a prenatally‐androgenized rat model of polycystic ovary syndrome in later life

**DOI:** 10.1113/EP091912

**Published:** 2024-11-29

**Authors:** Mahbanoo Farhadi‐Azar, Mahsa Noroozzadeh, Maryam Mousavi, Marzieh Saei Ghare Naz, Fahimeh Ramezani Tehrani

**Affiliations:** ^1^ Reproductive Endocrinology Research Center, Research Institute for Endocrine Sciences Shahid Beheshti University of Medical Sciences Tehran Iran; ^2^ Foundation for Research & Education Excellence Vestavia Hills AL USA

**Keywords:** glucose tolerance, insulin resistance, polycystic ovary syndrome (PCOS), prenatal androgen exposure, rat

## Abstract

Polycystic ovary syndrome (PCOS), one of the most common endocrine disorders in reproductive‐aged women, is associated with metabolic disturbances. The present study aimed to examine changes in body weight (BW) and glucose and insulin tolerance in a prenatally‐androgenized (PNA) rat model of PCOS compared to control with increasing age. Pregnant rats in the experimental group were subcutaneously injected with 5 mg of free testosterone on the 20th day of pregnancy, while the control group received the solvent. Female offspring of both groups, PNA rats (rat model of PCOS) and control, were examined in terms of changes in BW, glucose and insulin tolerance at 3, 6, 12 and 20 months of age. BW at birth (6.53 ± 0.89 vs. 5.60 ± 1.18 g; *P *= 0.038), 15 (25 ± 1.15 vs. 22.36 ± 3.98 g; *P *= 0.019) and 30 (59.37 ± 10.19 vs.49.9 ± 9.39 g; *P *= 0.022) days of age was significantly increased in the rat model of PCOS compared to control, but no significant differences were observed in BW of the rat model of PCOS compared to control at 60 (*P *= 0.155) and 75 (*P *= 0.932) days or at 3 (*P *= 0.239), 6 (*P *= 0.782), 12 (*P *= 0.755) and 20 (*P *= 0.092) months of age. Rat model of PCOS showed impaired glucose tolerance (IGT) at 3 months of age (*P *= 0.020) and insulin resistance (IR) with increasing age (3–20 months of age) compared to control. Increased BW before puberty, IGT at 3 months of age and IR with increasing age were observed in our rat model of PCOS. This rat model may contribute to a better understanding of underlying mechanisms of changes in BW, IGT and IR in future studies.

## Abstract (highlights)

- **What is the central question of this study?**
  Does increasing age affect glucose and insulin tolerance in a prenatally androgenized rat model of polycystic ovary syndrome (PCOS)?
- **What is the main finding and its importance?**
  Impaired glucose tolerance (IGT) at 3 months of age and insulin resistance (IR) with increasing age (3–20 months of age) were observed in our rat model of PCOS. This rat model of PCOS may contribute to a better understanding of underlying mechanisms of IGT and IR in future studies.

## 1 INTRODUCTION

Polycystic ovary syndrome (PCOS) is a complex endocrine disorder that affects 6–12% of reproductive‐aged women (Teede et al., 2010). It is characterized by hyperandrogenism, irregular ovulation and the presence of polycystic ovaries detected via ultrasound (Stener‐Victorin et al., 2024). PCOS is not only associated with reproductive manifestations but also linked to a wide range of metabolic disturbances, including insulin resistance (IR), dyslipidaemia, obesity, type 2 diabetes mellitus (T2DM) and cardiovascular diseases (CVDs) (Diamanti‐Kandarakis et al., 2006; Dunaif, 1997; Wild et al., 2010). These metabolic abnormalities affect reproductive function and overall health and well‐being of affected women in the long‐term (Cupisti et al., 2008; Kahal et al., 2018).

However, the exact aetiology of PCOS remains unclear, evidence suggests that a hyperandrogenic environment plays a central role in the development of PCOS (Chen & Pang, 2021; Kumar et al., 2022). Hormonal imbalances in the intrauterine environment can cause permanent genetic, physiological and morphological changes in fetal tissues and organs, leading to endocrine and reproductive disorders later in life (Barker, 2004; Skogen & Øverland, 2012). Studies have demonstrated that excessive androgen exposure in women with PCOS increases the risk of developing metabolic disorders such as IR, T2DM and dyslipidaemia (Armanini et al., 2022; Chen & Pang, 2021; Condorelli et al., 2018). These metabolic disorders can persist even after menopause and may contribute to an elevated risk of CVDs and other age‐related health problems (Jeong & Park, 2022; Macut et al., 2021; Stachowiak et al., 2015).

Several studies have investigated the metabolic disorders and their changes with age in PCOS patients (Escobar‐Morreale et al., 2005; Farhadi‐Azar et al., 2022; Kim et al., 2013; Moran et al., 2010). With ageing, women often experience changes in metabolic function that can increase the risk of developing metabolic disorders. These changes may include a decline in insulin sensitivity, alterations in lipid metabolism, and an increased predisposition to obesity and T2DM (Corpeleijn et al., 2009; Zhang et al., 2023). Additionally, the hormonal changes that occur during menopause can contribute to an increased risk of metabolic disturbances in women as they age (Stefanska et al., 2015).

Some studies have shown that women with PCOS have an increased risk of developing metabolic disorders and that metabolic abnormalities may persist even after menopause (Moran et al., 2010; Stachowiak et al., 2015). However, the results of these studies have been inconsistent, with some reporting improvements in metabolic parameters with increasing age, while others have shown no change or even worsening of metabolic profiles (Helvaci & Yildiz, 2020).

Human studies on this topic are scarce and face many challenges, such as loss to follow‐up in long‐term studies, being time consuming and costly, ethical issues, as well as the lack of possibility of certain investigations in humans, and therefore appropriate animal models of PCOS may contribute to a better understanding of this syndrome and any accompanying disorders. In our previous studies, we introduced a functional rat model of PCOS with similar features of PCOS to those of women, including hyperandrogenaemia, ovulatory dysfunction, disrupted oestrous cycle, increased body weight (BW), IR and hypertension at 3 months of age (Noroozzadeh et al., 2015; Sherman et al., 2018; Tehrani et al., 2014).

Considering the controversies regarding long‐term metabolic disorders in PCOS subjects, in the present study we aimed to investigate the long‐term metabolic disturbances (changes in BW, impaired glucose tolerance (IGT) and IR) in a prenatally‐ androgenized (PNA) rat model of PCOS compared to control.

## 2 METHODS

### 2.1 Ethical approval

The experiments described in this work were conducted in accordance with the ethics principles of laboratory animal care. The present study was approved by the institutional ethics review bord of the Research Institute for Endocrine Sciences (RIES) (approval reference number: IR SBMU ENDOCRINE.REC.1397.048). We followed established principles and practices to ensure the welfare of animals involved in this study and took all necessary steps to minimize their discomfort and suffering.

### 2.2 Animals and care

Ten virgin female Wistar rats, aged 75–95 days and weighing between 170 and 190 g, were obtained from the animal centre of RIES at Shahid Beheshti University of Medical Sciences, Tehran, Iran. Female rats were individually housed with male rats in polypropylene cages (43 × 30 × 15 cm) in a temperature‐controlled room (22 ± 3°C) with 12‐h light/dark cycles (lights on at 07.00 h) and a relative humidity of 45–55%. Food and water were provided ad libitum. The presence of a vaginal plug following mating marked the first day of pregnancy. The 10 pregnant rats were then randomly assigned to either the experimental or control (vehicle) group, with five rats in each group.

### 2.3 Prenatal testosterone treatment (inducing rat model of PCOS) and maintenance

To induce a rat model of PCOS, we used a method described in our previous study (Tehrani et al., 2014); briefly, five pregnant rats in the experimental group were subcutaneously injected with 5 mg free testosterone (T1500, 1 g, Sigma, Steinheim, Germany) dissolved in 500 µL of sesame oil (S3547, Sigma) and benzyl benzoate (B6630, Sigma) at a 4:1 ratio on gestational day 20. Simultaneously, each pregnant rat in the control group (*n* = 5) received 500 µL of the solvent (sesame oil and benzyl benzoate) using the same method (Tehrani et al., 2014). Based on our previous study, PNA female offspring were considered as the rat model of PCOS. In order to re‐confirm our rat model of PCOS, some of the most important features of PCOS such as total testosterone (TT) levels in blood serum, regularity in oestrous cycles and anogenital distance (AGD) were examined after puberty in PNA rats or the rat model of PCOS, and compared with their control (similar to our previous studies) (Noroozzadeh et al., 2015; Tehrani et al., 2014). A blood sample for TT measurement was collected from the tail of each rat. Blood sample was centrifuged at 6000 *g* for 5 min, at 4°C. TT levels were measured using an enzyme‐linked immunosorbent assay (ELISA) kit (Monobind, Inc., Lake Forest, CA, USA). Intra‐assay coefficients of variation for TT were <10%. At 70–80 days of age, the vaginal smears of PNA rats and control were checked daily between 08.00 and 10.00 h for 10 consecutive days to observe the changes in oestrous cycles. Collection and staining of vaginal smears were performed in the manner explained in our previous study (Tehrani et al., 2014). AGD (the distance (in millimeters) between the cranial edge of the anus and the base of the phallus) was measured using a vernier caliper.

**FIGURE 1:**
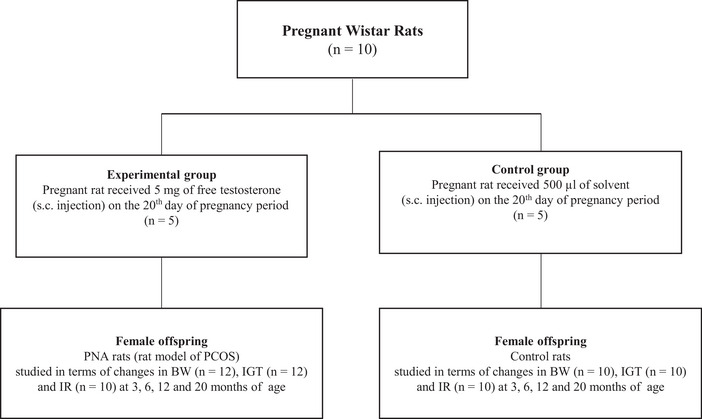
Study flowchart. Solvent: sesame oil and benzyl benzoate at a ratio of 4:1. BW, body weight; IGT, impaired glucose tolerance; IR, insulin resistance; PCOS, polycystic ovary syndrome; PNA rats, prenatally androgenized rats; s.c., subcutaneous.

For the present study, 12 rat models of PCOS and 10 control rats were housed separately in groups of three or four per cage with ad libitum food and water and all rats in both groups were assessed in terms of changes in BW, IGT and IR, in other words, glucose and insulin tolerance at 3, 6, 12 and 20 months of their age. A flowchart of the study is presented in Figure 1.

### 2.4 Measurement of BW in PCOS and control rats

BW of the rat model of PCOS and the control was measured at birth and at 15, 30, 45, 60 and 75 days of age, as well as at 3, 6, 12 and 20 months of age. BW was measured using a scale (Sartorius, Göttingen, Germany) with 1 g accuracy.

### 2.5 Intraperitoneal glucose tolerance test

During adulthood (3, 6, 12 and 20 months of age), an intraperitoneal glucose tolerance test (IPGTT) was conducted on the rat model of PCOS and the control. The test was performed after an overnight fast (12–14 h) between 08.00 and 11.00 h. Rats in both groups were anaesthetized using an intraperitoneal (i.p.) injection of pentobarbital sodium (P3761, 5 g, Sigma, St Louis, MO, USA) dissolved in 0.9% normal saline (45 mg kg^−1^ BW). Baseline (fasting) and subsequent blood samples were collected via tail cut. The baseline blood sample (0 min) was used to determine glucose and insulin concentrations. Glucose (2 g kg^−1^ BW) was administered through an i.p. injection, and blood samples were collected via tail cut at 10, 20, 30, 60 and 120 min after glucose administration.

### 2.6 Intraperitoneal insulin tolerance test

Three days after IPGTT at each time point of animal age (3, 6, 12 and 20 months of age) an intraperitoneal insulin tolerance tests (IPITT) was conducted on the rat model of PCOS and the control. The tests were performed after an overnight fast (12–14 h) between 08.00 and 11.00 h. Rats in both groups were anaesthetized using an i.p. injection of pentobarbital sodium (45 mg kg^−1^ BW). Baseline (fasting) and subsequent blood samples were collected via tail cut. Recombinant (recombinant DNA origin) regular human insulin (0.75 U kg^−1^ BW) was administered through an i.p. injection, and blood samples were collected via a tail cut before (0 min or baseline) and 10, 20, 30, 40 and 60 min after insulin administration. After completing the IPITT at 20 months of age, rats were sacrificed by heart incision while they were deeply anaesthetized with pentobarbital sodium (45 mg kg^−1^ BW). Loss to follow‐up happened for two rat models of PCOS, and IPITT data were collected for 10 rat models of PCOS.

Blood samples (0.3 mL at each time point for IPGTT and IPITT) were centrifuged at 4°C and 6000 *g* for 5 min. The sera were stored at −80° C for subsequent measurement of glucose and insulin concentrations.

### 2.7 Assessment of serum glucose and insulin concentrations

Serum glucose (Pars Azmoon Co., Tehran, Iran) and insulin (Zellbio, Lonsee, Germany, cat. no. ZB‐10707C‐R9648) concentrations were determined using the glucose oxidase method and the ELISA method, respectively. Intra‐assay coefficients of variation for glucose and insulin measurements were <10%. The detection limits were 5–400 mg/dL and 1.5–48 mIU/L for glucose and insulin, respectively, and sensitivity for insulin was 0.2 mIU/L.

### 2.8 Homeostasis model assessment of β‐cell function

Baseline serum samples before a glucose tolerance test (GTT) (0 min) were used to determine fasting glucose and insulin concentrations. A homeostasis model assessment of β‐cell function (HOMA‐β) was calculated using the following formula: HOMA‐β = (20 × insulin/glucose − 3.5)%, where glucose and insulin are given in mmol/L and mIU/L, respectively.

### 2.9 Statistical analysis

For the continuous variables, we conducted the Kolmogorov–Smirnov normality test. Data for BW are presented as means ± standard deviation (SD). An independent Student's *t*‐test was used to compare the results of BW between the rat model of PCOS and control.

Furthermore, the generalized estimating equation (GEE) model was used to evaluate secular longitudinal trends of serum glucose concentrations during the IPGTT and IPITT in the rat model of PCOS and control based on repeated times for ages 3, 6, 12 and 20 months, separately (Liang & Zeger, 1986). Also, the secular longitudinal trends of IPGTT, IPITT and HOMA‐β in two groups at four ages (3, 6, 12 and 20 month) based on repeated times were evaluated by the GEE model. GEE analysis considers correlations within subjects using a working correlation matrix. In situations where missing data or incomplete follow‐up is a concern, the GEE method is a more appropriate statistical approach; it utilizes all available data for each subject (each rat) at the various time points, thereby mitigating the limitations associated with repeated measures ANOVA. The mean changes trend plot was used to describe the changes in means of serum glucose concentrations during the IPGTT and IPITT at repeated ages.

To ensure the adequacy of our sample size for GEE, we conducted a *post hoc* power analysis using simulation‐based methods. We simulated datasets based on our study design and parameters, fitting GEE models to each simulated dataset. The proportion of simulations in which the null hypothesis was rejected provided an estimate of the power of our study.

The estimated effect size for the difference in GTT and insulin tolerance test (ITT) trends between the PCOS and control groups was considered 0.5 and α (type 1 error) was considered 0.05. The *post hoc* power analysis indicated that our study had a power of approximately 0.84 (84%) to detect this effect size, given the sample size and the presence of missing data.

Statistical analysis was performed using the software package STATA (version 12; STATA Inc., College Station, TX, USA). A *P*‐value < 0.05 was considered statistically significant.

## 3 RESULTS

### 3.1 Re‐confirmation of our rat model of PCOS

TT levels were significantly elevated in the rat model of PCOS compared to control (1.40 ± 0.3 vs. 1.007 ± 0.24 ng/mL, *P* = 0.003) during the oestrus phase of the oestrous cycle. Examination of vaginal smears over a consecutive period of 10 days revealed that the rat model of PCOS exhibited longer and more irregular oestrous cycles compared to control. The presence of a combination of epithelial and cornified cells in the vaginal smears along with an extended oestrous cycle lasting more than 4–5 days was observed in the rat model of PCOS. Additionally, AGD significantly increased in the rat model of PCOS compared to control (17.58 ± 1.16 vs. 11.70 ± 1.05 mm; *P* < 0.001).

### 3.2 BW

**FIGURE 2:**
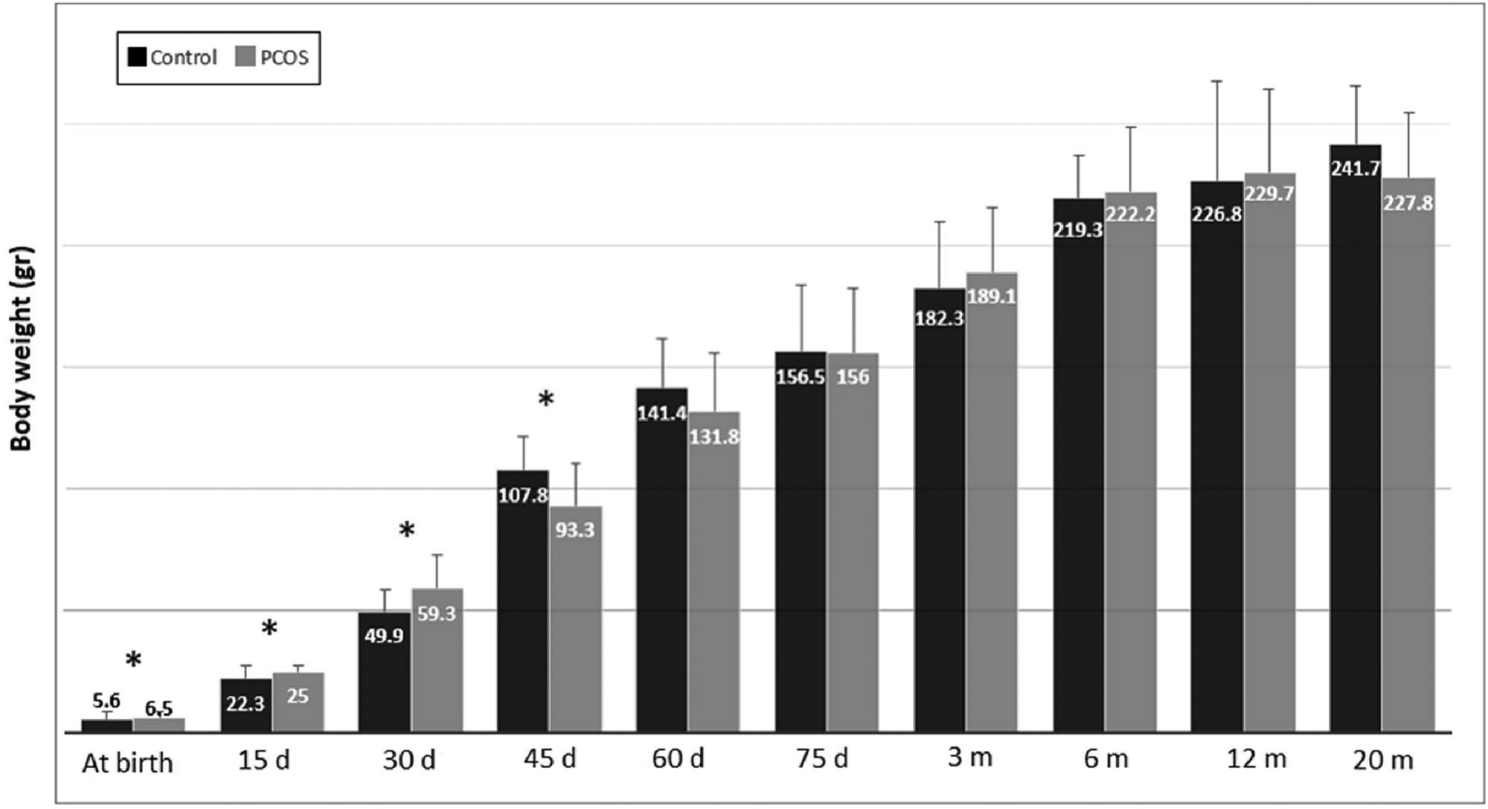
Comparison of mean body weight (BW) in the rat model of PCOS (*n* = 12) and control (*n* = 10) at different ages. Values are expressed as means (SD). An independent Student's *t*‐test was used to compare the results between the two groups. *P < 0.05. d, days; m, months; PCOS, polycystic ovary syndrome.

Comparison of BW in the rat model of PCOS compared to control at different ages is presented in Figure 2. BW of the rat model of PCOS at birth (6.53 ± 0.89 vs. 5.60 ± 1.18 g; *P* = 0.038), 15 (25 ± 1.15 vs. 22.36 ± 3.98 g; *P* = 0.019) and 30 (59.37 ± 10.19 vs. 49.9 ± 9.39 g; *P* = 0.022) days of age was significantly higher than control; however, at 45 days of age, BW in the rat model of PCOS was significantly lower than control (93.31 ± 15.83 vs. 107.87 ± 10.57 g; *P* = 0.029). On the other hand, no significant differences were observed at 60 and 75 days of age or at 3, 6, 12 and 20 months of age between the two groups (rat model of PCOS and control) (*P* > 0.05).

### 3.3 IPGTTs at 3, 6, 12 and 20 months of age in the rat model of PCOS compared to control

**FIGURE 3:**
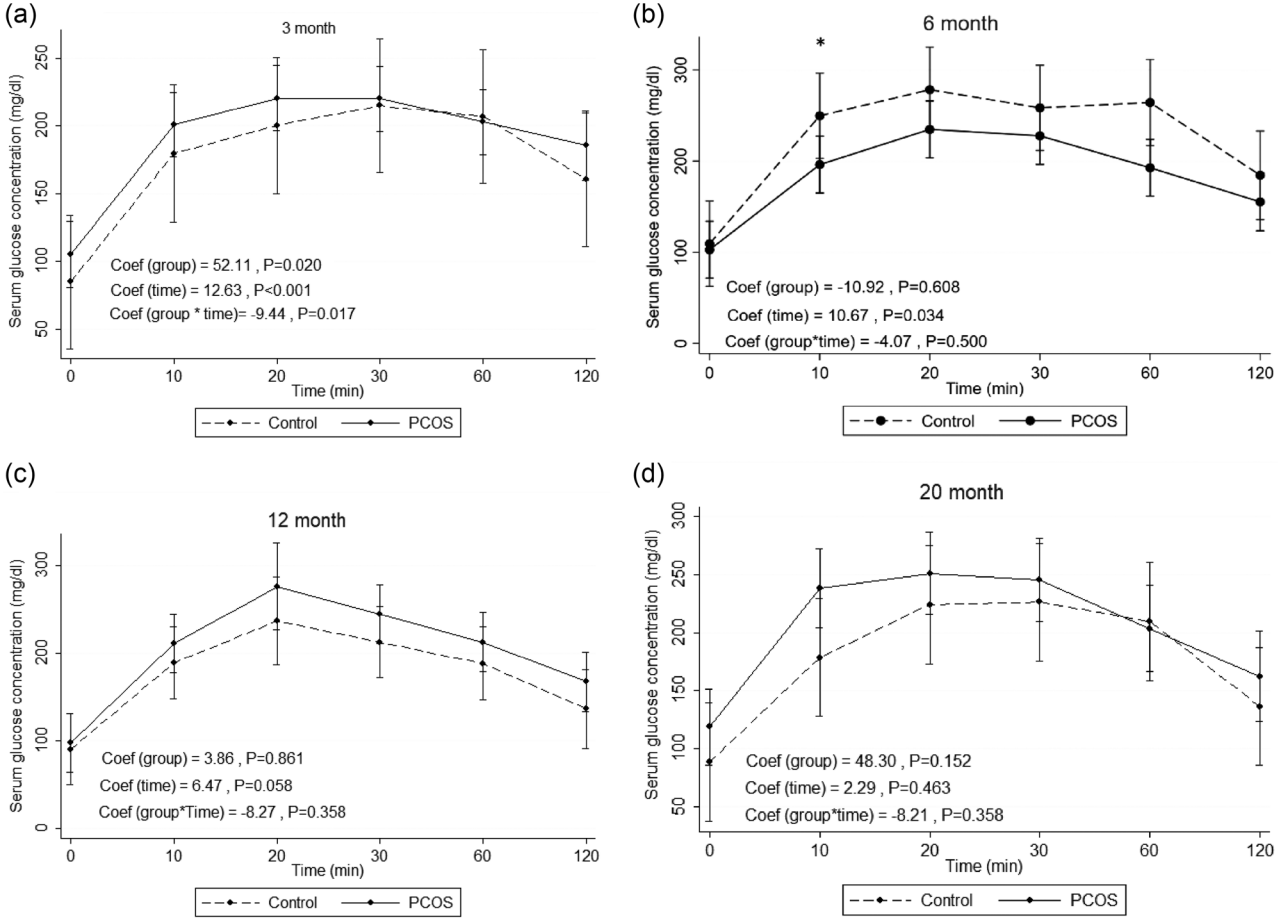
IPGTT: mean change of serum glucose concentration during intraperitoneal glucose tolerance test in the rat model of PCOS and control at 3 (a), 6 (b), 12 (c) and 20 (d) months of age. PCOS, *n* = 12; control, *n* = 10. Generalized estimating equation (GEE) measures. Mean change of serum glucose concentration (mg/dL) within repeated times (min) between two groups of rats (rat model of PCOS and control), assuming the interaction between time and group status. Coef, coefficient; IPGTT, intraperitoneal glucose tolerance test; PCOS, polycystic ovary syndrome.

Table 1 shows the results of GEE models to estimate the effect of PCOS compared to control on the trends of means for glucose concentration based on their age. At 3 months of age, mean change of serum glucose concentration was significantly higher in the rat model of PCOS than in control (52.11; 95% CI: 85.35, 95.87; *P* = 0.020). This finding possibly indicates IGT at this time point of age. However, no significant differences were observed between the two groups at other ages (6, 12 and 20 months). Figure 3 illustrates the trends of means for glucose concentration in the rat model of PCOS and control based on their age.

**TABLE 1:** Mean (95% CI) change of glucose concentration in IPGTT in the rat model of PCOS compared to control: results from GEE analysis.

	Coefficient (95% CI)	*P*
**3** **months**		
Group	52.11 (8.35, 95.87)	**0.020**
Time	12.63 (5.60, 19.66)	**<0.001**
Group × Time	−9.44 (−17.23, −1.65)	**0.017**
**6** **months**		
Group	−10.92 (−52.70, 30.85)	0.608
Time	10.67 (0.78, 20.56)	**0.034**
Group × Time	−4.07 (−15.90, 7.75)	0.500
**12** **months**		
Group	3.86 (−39.34, 47.07)	0.861
Time	6.47 (−0.21, 13.17)	0.058
Group × Time	5.91 (−2.29, 14.11)	0.158
**20** **months**		
Group	48.30 (−17.70, 114.31)	0.152
Time	5.29 (−8.83, 19.41)	0.463
Group × Time	−8.27 (−25.90, 9.36)	0.358

*Note*: Control group was considered as a reference group. *P*‐values shown in bold indicate statistical significance. PCOS, *n* = 12; control, *n* = 10. Abbreviations: CI, confidence interval; GEE, generalized estimating equation; IPGTT, intraperitoneal glucose tolerance test; PCOS, polycystic ovary syndrome.

### 3.4 Serum glucose concentrations at baseline and different time points of IPGTT in the rat model of PCOS compared to control with increasing age

**FIGURE 4:**
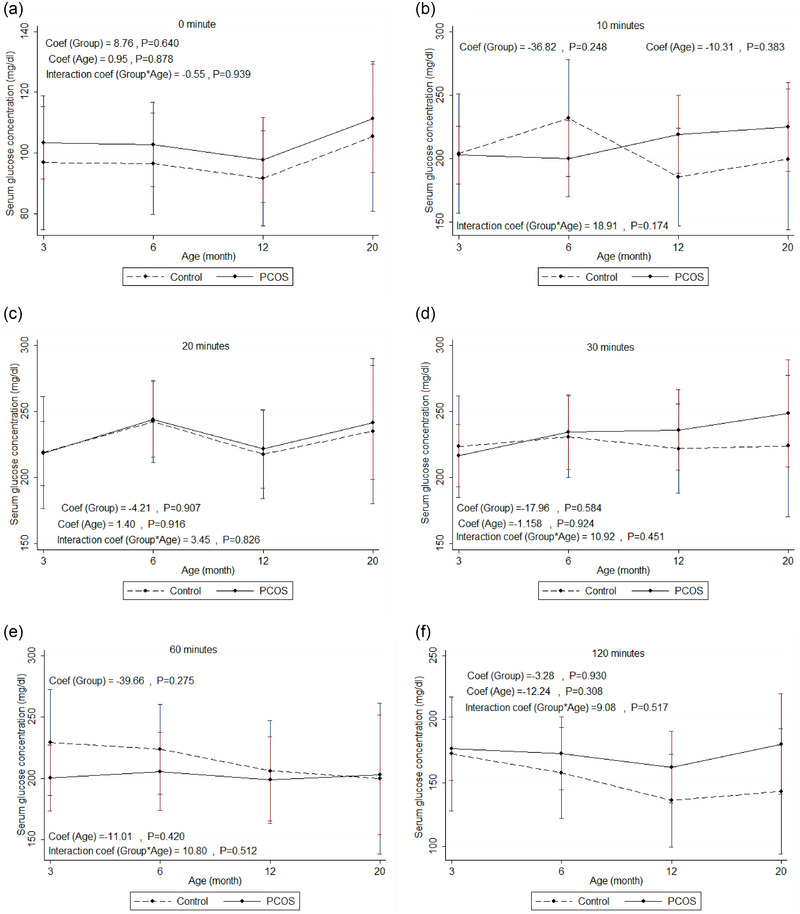
IPGTT: mean change of serum glucose concentration at baseline and different time points (a–f) of IPGTT in the rat model of PCOS compared to control with increasing age (from 3 months to 20 months of age). PCOS, *n* = 12; control, *n* = 10. Generalized estimating equation (GEE) measures. Mean change of serum glucose concentration (mg/dL) within repeated ages (months) between two groups of rats (rat model of PCOS and control), assuming the interaction between age and group status. Coef, coefficient; IPGTT, intraperitoneal glucose tolerance test; PCOS, polycystic ovary syndrome.

Table 2 and Figure 4 show mean changes of serum glucose concentration at baseline and different time points of IPGTT in the rat model of PCOS compared to control with increasing age (from 3 to 20 months of age). The results showed no statistically significant differences between mean change for glucose concentration in the rat model of PCOS compared to control with increasing age at baseline and any time point of IPGTT (10, 20, 30, 60 and 120 min after glucose administration).

**TABLE 2:** Mean (95% CI) change of glucose concentration at baseline and different time points of IPGTT in the rat model of PCOS compared to control with increasing age (3–20 months of age): results from GEE analysis.

	Coefficient (95% CI)	*P*
**0 (baseline)**		
Group	8.76 (−27.9, 45.42)	0.640
Time	0.95 (−11.32, 13.23)	0.878
Group × Time	−0.55 (−14.75, 13.63)	0.939
**10** **min**		
Group	−36.82 (−99.32, 25.67)	0.248
Time	−10.31 (−33.51, 12.87)	0.383
Group × Time	18.91 (−8.34, 46.17)	0.174
**20** **min**		
Group	−4.21 (−75.19, 66.77)	0.907
Time	1.40 (−24.51, 27.31)	0.916
Group × Time	3.45 (−27.38, 34.28)	0.826
**30** **min**		
Group	−17.96 (−82.20, 46.27)	0.584
Time	−1.15 (−24.95, 22.63)	0.924
Group × Time	10.92 (−17.45, 39.30)	0.451
**60** **min**		
Group	−39.66 (−110.94, 31.60)	0.275
Time	−11.01 (−37.78, 15.74)	0.420
Group × Time	10.80 (−21.45, 43.05)	0.512
**120** **min**		
Group	−3.28 (−76.95, 70.39)	0.930
Time	−12.24 (−35.79, 11.30)	0.308
Group × Time	9.08 (−18.38, 36.55)	0.517

*Note*: Control group was considered as a reference group. PCOS, *n* = 12; control, *n* = 10. Abbreviations: CI, confidence interval; GEE, generalized estimating equation; IPGTT, intraperitoneal glucose tolerance test; PCOS, polycystic ovary syndrome.

### 3.5 IPITTs at 3, 6, 12, and 20 months of age in the rat model of PCOS compared to control

**FIGURE 5:**
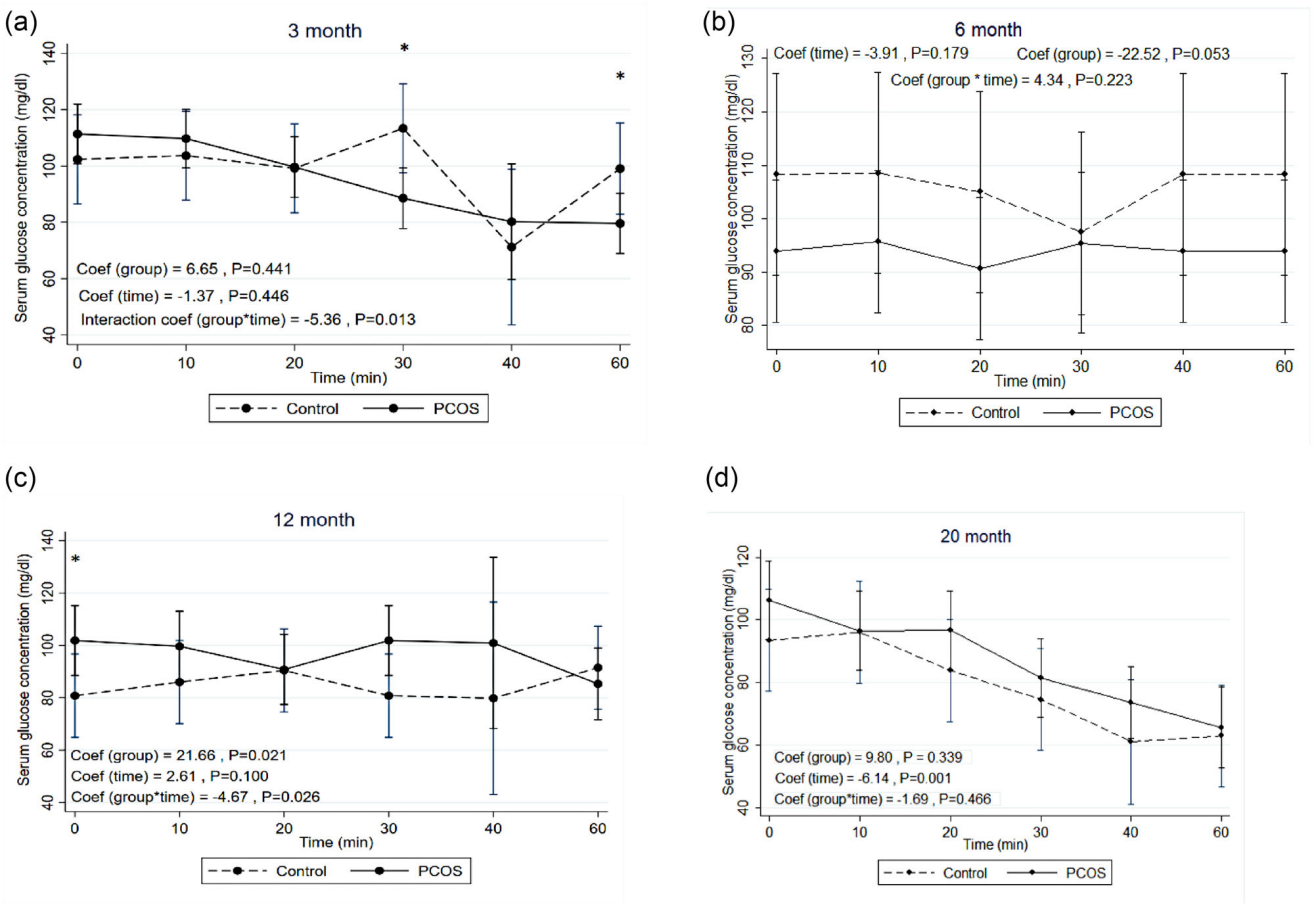
IPITT: mean change of serum glucose concentration during IPITT in the rat model of PCOS and control at 3 (a), 6 (b), 12 (c), and 20 (d) months of ages. PCOS, *n* = 10; control, *n* = 10. Generalized estimating equation (GEE) measures. Mean change of serum glucose concentration (mg/dL) within repeated times (min) between two groups of rats (rat model of PCOS and control), assuming the interaction between time and group status. Coef, coefficient; IPITT, intraperitoneal insulin tolerance test; PCOS, polycystic ovary syndrome.

Table 3 shows the results of GEE models to estimate the effect of PCOS compared to control on the trends of means for glucose concentration based on the age of the rats. At 12 months of age, mean change of serum glucose concentration was significantly higher in the rat model of PCOS than in control (21.66; 95% CI: 3.32, 40.01; *P* = 0.021); this result possibly indicates IR in the rat model of PCOS at this age. On the other hand, there were no significant differences between the two groups at other ages. Figure 5 presents the trends of means for glucose concentration in the rat model of PCOS and control based on their age.

**TABLE 3:** Mean (95% CI) change of glucose concentration in IPITT in the rat model of PCOS compared to control: results from GEE analysis.

	Coefficient (95% CI)	*P*‐value
**3** **months**		
Group	6.65 (−10.26, 23.56)	0.441
Time	−1.37 (−4.90, 2.15)	0.446
Group × Time	−5.36 (−9.59, −1.13)	**0.013**
**6** **months**		
Group	−22.52 (−45.33, 0.29)	0.053
Time	−3.91 (−9.61, 1.78)	0.179
Group × Time	4.34 (−2.63, 11.32)	0.223
**12** **months**		
Group	21.66 (3.32, 40.01)	**0.021**
Time	2.61 (−0.50, 5.74)	0.100
Group × Time	−4.67 (−8.79, −0.55)	**0.026**
**20** **months**		
Group	9.80 (−10.30, 29.92)	0.339
Time	−6.14 (−9.69, −2.59)	**<0.001**
Group × Time	−1.69 (−6.25, 2.86)	0.466

*Note*: Control group was considered as a reference group. PCOS, *n* = 10; control, *n* = 10. *P*‐values shown in bold indicate statistical significance. Abbreviations: CI, confidence interval; GEE, generalized estimating equation; IPITT, intraperitoneal insulin tolerance test; PCOS, polycystic ovary syndrome.

### 3.6 Serum glucose concentrations at baseline and different time points of IPITT in the rat model of PCOS compared to control with increasing age

**FIGURE 6:**
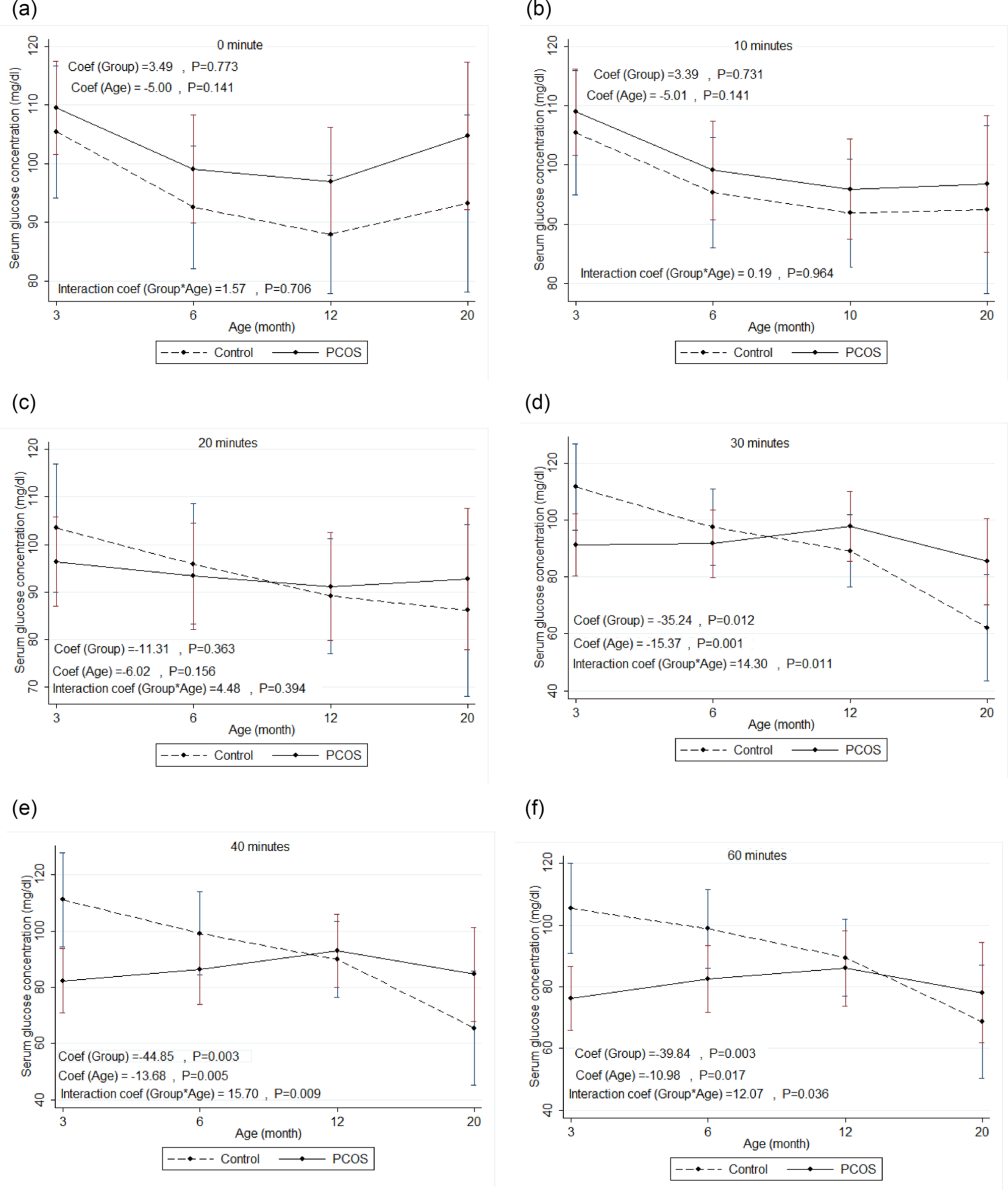
IPITT: mean change of serum glucose concentration at baseline and different time points (a–f) of IPITT in the rat model of PCOS compared to control with increasing age (from 3 months to 20 months of age). PCOS, *n* = 10; control, *n* = 10. Generalized estimating equation (GEE) measures. Mean change of serum glucose concentration (mg/dL) within repeated ages (month) between two groups of rats (rat model of PCOS and control), assuming the interaction between age and group status. Coef, coefficient; IPITT, intraperitoneal insulin tolerance test; PCOS, polycystic ovary syndrome.

Table 4 and Figure 6 show mean changes of serum glucose concentration at baseline and different time points of IPITTs in the rat model of PCOS compared to control with increasing age (from 3 months to 20 months of age). The results showed no statistically significant differences between mean change for glucose concentration in the rat model of PCOS compared to control with increasing age at baseline and time points 10 and 20 min after insulin administration. The mean changes for serum glucose concentration at 30 (−35.24; 95% CI: −62.69, −7.79; *P* = 0.012), 40 (−44.85; 95% CI: −74.93, −14.78; *P* = 0.003) and 60 (−39.84; 95% CI: −66.14, −13.55; *P* = 0.003) min after insulin injection during IPITT in the rat model of PCOS were significantly lower than their control with increasing age, possibly representing IR in the rat model of PCOS with increasing age.

**TABLE 4:** Mean (95% CI) change of glucose concentration at baseline and different time points of IPITT in the rat model of PCOS compared to control with increasing age (3–20 months of age): results from GEE analysis.

	Coefficient (95% CI)	*P*
**0 (baseline)**		
Group	3.49 (−16.58, 23.57)	0.733
Time	−5.00 (−11.66, 1.65)	0.141
Group × Time	1.57 (−6.62, 9.78)	0.706
**10** **min**		
Group	3.39 (−15.95, 22.74)	0.731
Time	−5.01 (−11.69, 1.65)	0.141
Group × Time	0.19 (−8.05, 8.43)	0.964
**20** **min**		
Group	−11.31 (−35.68, 13.06)	0.363
Time	−6.02 (−14.33, 2.29)	0.156
Group × Time	4.48 (−5.82, 14.79)	0.394
**30** **min**		
Group	−35.24 (−62.69, −7.79)	**0.012**
Time	−15.37 (−24.30, −6.44)	**<0.001**
Group × Time	14.30 (3.31, 25.29)	**0.011**
**40 min**		
Group	−44.85 (−74.93, −14.78)	**0.003**
Time	−13.68 (−23.26, −4.11)	**0.005**
Group × Time	15.70 (3.89, 27.51)	**0.009**
**60** **min**		
Group	−39.84 (−66.14, −13.55)	**0.003**
Time	−10.98 (−19.98, −1.99)	**0.017**
Group × Time	12.07 (0.81, 23.33)	**0.036**

*Note*: Control group was considered as a reference group. PCOS, *n* = 10; control, *n* = 10. *P*‐values shown in bold indicate statistical significance. Abbreviations: CI, confidence interval; GEE, generalized estimating equation; IPITT Intraperitoneal insulin tolerance test; min, minute; PCOS, polycystic ovary syndromes.

### 3.7 HOMA‑β index

**FIGURE 7:**
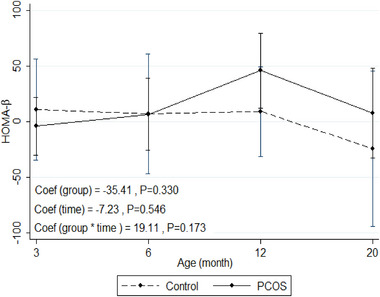
HOMA‐β: mean change of HOMA‐β in the rat model of PCOS compared to control with increasing age (from 3 to 20 months of age). PCOS, *n* = 12; control, *n* = 10. Generalized estimating equation (GEE) measures. Mean change of HOMA‐β within repeated ages (month) between two groups of rats (rat model of PCOS and control), assuming the interaction between age and group status. Coef, coefficient; HOMA‐β, homeostasis model assessment of β‐cell function; PCOS, polycystic ovary syndrome.

Figure 7 shows the mean change of HOMA‐β (a useful index of β‐cell function) in the rat model of PCOS compared to control with increasing age (from 3 to 20 months of age). GEE model results showed no significant difference in the trend of mean changes for the values of HOMA‐β with increasing age between the two groups.

## 4 DISCUSSION

In the present study, our PNA rat model of PCOS exhibited increased BW before puberty, IGT at 3 months of age and IR with increasing age (3 to 20 months of age). In agreement with previous studies conducted on humans and animals (Abbott et al., 2005; Amalfi et al., 2012; Noroozzadeh et al., 2015; van Houten et al., 2012; Yan et al., 2013), IGT and IR were observed in our rat model of PCOS.

IR is a condition in which the cells of the body do not respond effectively to insulin, and as a result, the level of insulin in the blood increases (Dunaif, 1997). IGT is a condition in which the body has difficulty processing glucose effectively, leading to elevated blood sugar levels (Moran et al., 2010). In patients with PCOS, IR can contribute to IGT by impairing the body's ability to respond appropriately to insulin. This can lead to a range of metabolic disorders, including an increased risk of diabetes and other CVD risk factors (Dunaif, 1997; Palmert et al., 2002). In general, the mechanisms of IGT and IR in patients with PCOS are complex, and a combination of hyperandrogenism, obesity, chronic inflammation, insulin signalling abnormalities and genetic factors contribute to the development of IGT and IR in patients with PCOS (Armanini et al., 2022; Dunaif, 1997; Hernández‐Jiménez et al., 2022; Macut et al., 2017).

In a study of obese adolescents with PCOS, only one patient had IR (Bridger et al., 2006), while other studies of obese adolescents with PCOS reported IGT rates of 33–52% (Arslanian et al., 2001; Palmert et al., 2002). In contrast, non‐obese adolescents with PCOS have not been reported to have an increased risk of IGT (Silfen et al., 2003), suggesting a direct relationship between glucose tolerance (GT) and obesity. IR has a direct relationship with PCOS and is one of the most important metabolic disorders in PCOS subjects (Demissie et al., 2008). The prevalence of IR is reported to be approximately 50–70% in women with PCOS (Dunaif, 1997), which can reach 95% in obese women (Carmina et al., 2004).

Women with PCOS often show defects in insulin signalling pathways, including reduced expression of insulin receptors or downstream signalling molecules. These abnormalities can lead to a decrease in insulin sensitivity and disruption of glucose absorption by cells (Diamanti‐Kandarakis et al., 2006; Li et al., 2022).

Phosphoinositide 3‐kinases (PI3K)–AKT (protein kinase B) signalling is one of the major pathways for insulin signalling. Insulin induces the PI3K–AKT pathway and mediates its regulatory effects on metabolism (Zhang et al., 2019). The AKT complex can translocate glucose transporters, including glucose transporter type 4 (GLUT4), to the cell membrane, thereby increasing glucose uptake and lowering blood glucose levels (Zhang et al., 2019). In contrast, mitogen‐activated protein kinase (MAPK) activation with reduced GLUT4 expression and impaired glucose transport is associated with the development of IR (Zhou et al., 2018), and glucose levels are impaired by AKT2 deficiency due to the inability of insulin to activate signalling pathways (Cho et al., 2001). In addition to cellular mechanisms, there are potential mechanisms through which metabolic perturbations in early life may have long‐term effects on age‐related outcomes. One possibility is that prenatal androgen exposure during fetal development may program the metabolic system to be more sensitive to GT and IR later in life. This is consistent with the concept of embryonic programming, which suggests that prenatal androgen exposure during developmental periods can have long‐lasting effects on physiological processes (Roland et al., 2010).

Thus, our findings raise the possibility that excess androgen exposure during prenatal life may influence peripheral insulin sensitivity in PCOS through other mechanisms. One of the most important mechanisms involved in IR in PCOS is increased phosphorylation of a serine residue of insulin receptor substrate 1 (IRS‐1) in the insulin signalling pathway (Corbould et al., 2006). One study shows that IRS‐1‐related PI3K activity is significantly decreased in PCOS, which indicates impaired insulin response in skeletal muscle and IR (Dunaif et al., 2001). Furthermore, in another study, a rat model of PCOS showed decreased insulin‐stimulated AktB in skeletal muscle as well as decreased abundance of IRS‐1 and IRS‐2 in skeletal muscle and liver (Yan et al., 2013), leading to IR. The results of the present study are consistent with previous studies that have shown that PCOS is associated with IGT and IR (Krstevska et al., 2021; Moran et al., 2010).

Several studies have investigated the links between androgen excess exposure and the metabolic profile in PCOS subjects (Kempegowda & Melson, 2020; Sanchez‐Garrido & Tena‐Sempere, 2020); understanding these links is essential for developing targeted therapies for those women affected by PCOS. For instance, treatments that address both hyperandrogenism and IR may help mitigate the associated metabolic complications (Asimakopoulos et al., 2024; Fedeli et al., 2024; Genazzani et al., 2008).

Studies have shown that prenatal androgen exposure directly impairs pancreatic islet and β‐cell function, thereby impairing insulin secretion. Exposure of isolated pancreatic islets to androgens in vitro showed an impaired response to glucose stimulation, suggesting that androgens may have both activating and organizational effects on pancreatic islet function (Roland et al., 2010).

Previous studies showed that patients with PCOS often show abnormalities in β‐cell function that contribute to IR and IGT. Findings show that abnormalities in β‐cell function play an important role in the pathogenesis of metabolic disorders in PCOS (Cerf, 2013; Goodarzi et al., 2005). Our study demonstrated a rising trend in β‐cell function compared to control. This may be associated with increased insulin secretion in the rat model of PCOS as a compensatory mechanism for IR and to maintain glucose homeostasis in response to IGT.

### 4.1 Strengths and limitations of the study

The primary strength of this investigation lies in its novel approach, as it is one of the first studies to comprehensively examine metabolic disturbances, including IGT and IR, in a PNA‐ rat model of PCOS compared to an age‐matched control across various age intervals. Another strength of our study is our rat model of PCOS (PNA‐rat model of PCOS) used in the present study. Various prenatal and postnatal animal models of PCOS have been developed to date (Basak et al., 2024; Noroozzadeh et al., 2017; Stener‐Victorin et al., 2020). However, prenatal models are generally preferred, as they provide permanent features of PCOS, in contrast to the temporary characteristics observed in postnatal models (Noroozzadeh et al., 2017). Among the various prenatal PCOS rat models, our model, which involves a single dose of testosterone administered on the 20th day of fetal life (Tehrani et al., 2014; Noroozzadeh et al., 2015), is favoured over those that administer androgen during days 15–19 of fetal life (Chinnathambi et al., 2012; Sathishkumar, Elkins, Chinnathambi et al., 2011; Sathishkumar, Elkins, Yallampalli et al., 2011; Tehrani et al., 2014). The latter approach has been associated with developmental and morphological abnormalities in the reproductive system and androgen‐sensitive tissues that are not typically observed in PCOS (Tehrani et al., 2014; Wu et al., 2010). Furthermore, a single injection minimizes stress in the animals and may be regarded as more ethically acceptable.

On the other hand, our study has some limitations, it is essential to acknowledge these constraints. Specifically, our analysis did not include assessments of sex steroid hormones and lipids, which could have provided valuable insights into the metabolic profile of the rat model of PCOS. An extended fasting period prior to the ITT in rats, may indeed pose potential issues such as stress, metabolic changes, hypoglycaemia risk and altered hormonal responses, as well as ethical considerations. However, this duration is standard for these tests and has been utilized in previous studies involving an ITT (Gheibi et al., 2017; Liu et al., 2018). Typically, rodents (rats and mice) are fasted overnight for 16–18 h, which is consistent with established protocols for rodents undergoing an ITT. Another consideration is the potential for pseudoreplication in our study. However, we believe that the impact of pseudoreplication on our results is minimal because the multiple glucose measurements taken during the IPGTT and IPITT at distinct time points after glucose or insulin administration do not constitute repetitions of the same test. Instead, each rat underwent repeated assessments of glucose values during the IPGTT or IPITT, but these measurements were taken at distinct time intervals, thereby minimizing the risk of pseudoreplication.

### 4.2 Conclusion

Increased BW before puberty, IGT at 3 months of age and IR with increasing age were observed in our PNA‐rat model of PCOS. This rat model of PCOS may contribute to better understanding of underlying mechanisms of changes in BW, IGT and IR in future studies.

## AUTHOR CONTRIBUTIONS

Fahimeh Ramezani Tehrani and Mahsa Noroozzadeh did the study design and supervised the whole project. Mahbanoo Farhadi‐Azar and Mahsa Noroozzadeh conducted hands‐on experiments for data collection. Maryam Mousavi analyzed data and performed statistical analyses. Mahbanoo Farhadi‐Azar wrote the original draft. Mahbanoo Farhadi‐Azar, Fahimeh Ramezani Tehrani, Mahsa Noroozzadeh, Maryam Mousavi, and Marzieh Saei Ghare Naz revised and finalized the manuscript. All authors have read and approved the final version of this manuscript and agree to be accountable for all aspects of the work in ensuring that questions related to the accuracy or integrity of any part of the work are appropriately investigated and resolved. All persons designated as authors qualify for authorship, and all those who qualify for authorship are listed.

## CONFLICT OF INTEREST

The authors declare no conflicts of interest.

## Data Availability

Supporting data are available upon request.
